# Measuring intraocular pressure

**Published:** 2019-12-17

**Authors:** Elmien Wolvaardt, Sue Stevens

**Affiliations:** 1 Editor: *Community Eye Health Journal*, International Centre for Eye Health, London School of Hygiene & Tropical Medicine, UK.; 2 Former Nursing Advisor: *Community Eye Health Journal*, International Centre for Eye Health, London School of Hygiene & Tropical Medicine, UK.

**High intraocular pressure (IOP) is an important warning sign. Left untreated, it can result in irreversible damage to the optic nerve. Patients with suspected high IOP must be referred to an ophthalmologist for a detailed and comprehensive eye examination.**

**Figure 1:**
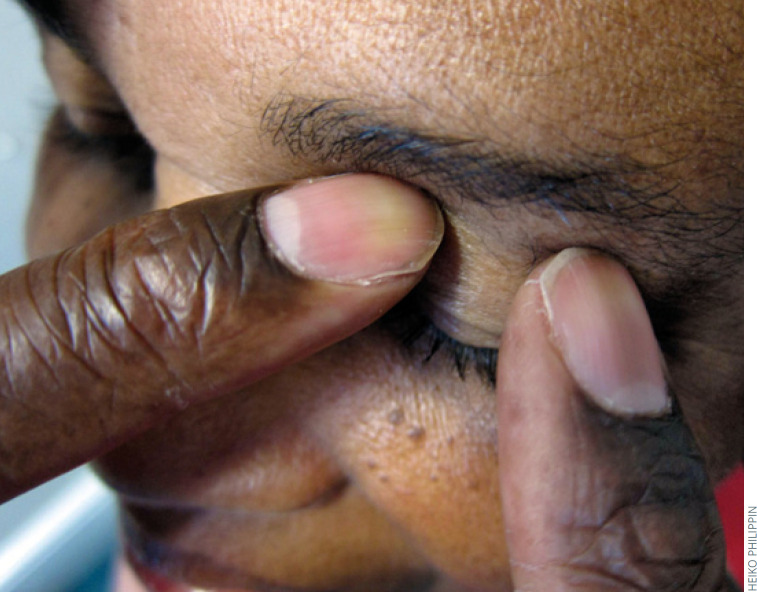
Palpating the eye carefully can help to identify very high intraocular pressure, a possible sign of glaucoma. TANZANIA

Normal intraocular pressure (IOP) ranges from 12–22 mm Hg, on average, but it may be higher if patients have glaucoma, use medication (e.g. steroids) or have recently undergone eye surgery.

The International Agency for the Prevention of Blindness (IAPB) recommends that trained ophthalmic personnel measure IOP using either a Perkins tonometer (used for applanation tonometry) or new technologies such as puff tonometers or the Tonopen.^1^

If these are not available, and if the patient's history or symptoms suggest that the IOP may be high, there are two screening tests that may be useful:

1. The fingertip test (digital palpation)
2. Schiotz tonometry

**Figure 2:**
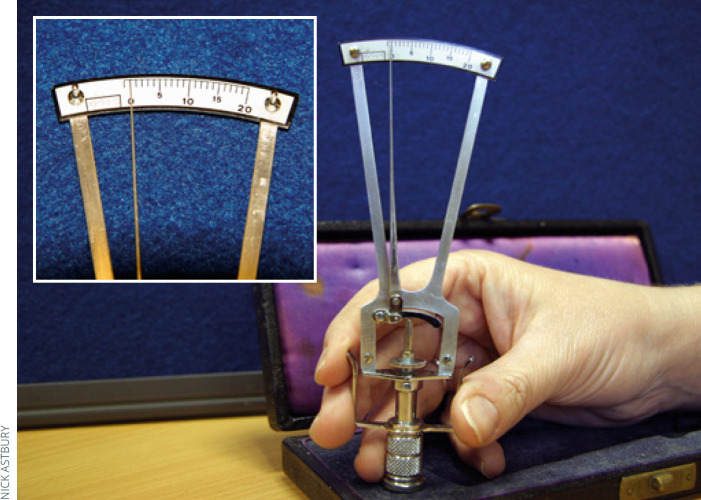
Schiötz tonometer. The pointer should be at ‘0’ when using the 5.5 g weight

## 1. The fingertip test

It is possible to detect very high IOP using your fingertips. The accuracy is better if the examiner is familiar with this examination method, so take time to practice it: first on yourself and then on your colleagues (with their permission).

**Note:** If you do not detect anything abnormal, the eye pressure may still be dangerously high. If the history or symptoms suggest glaucoma, or if the patient is using steroid medication or has recently undergone eye surgery, you must refer them to a centre where their IOP can be accurately assessed.

### Method^2^

- Ask the patient to close her or his eyes and look down.
- Place the tips of both index fingers on the closed upper eyelid. Keeping both fingertips in contact with the upper eyelid, apply gentle pressure through the closed eyelid, first gently pressing on the eye with the right index finger, then with the left, and then with the right again (Figure 1).
- Repeat on the other eye.
- A normal eye should feel a bit like a tomato that is just ripe: not solid, nor very soft.
- It is important to compare the two eyes with one other. An eye with very high IOP will feel abnormally hard and solid.^3^

## 2. Schiötz tonometry^4^

Schiötz tonometry is a more accurate screening test. If Schiötz tonometry indicates a high IOP, the patient should be referred to an ophthalmologist who will be able to confirm the result (using applanation tonometry or equivalent) and begin appropriate management.

**Figure 3:**
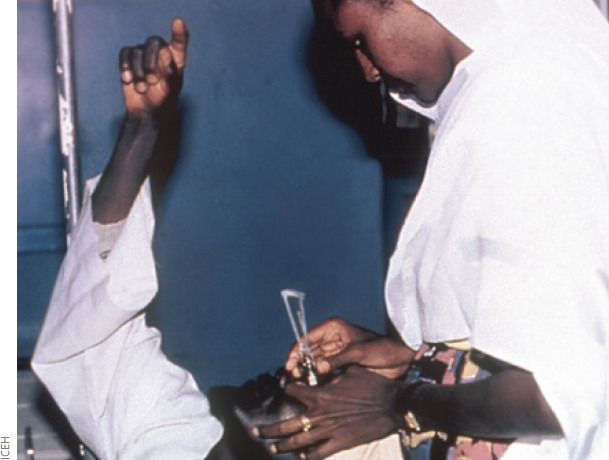
Position yourself behind the patient with your hands at the same level as their head. The patient is looking at his finger, which is directly above his eyes.

**Figure 4:**
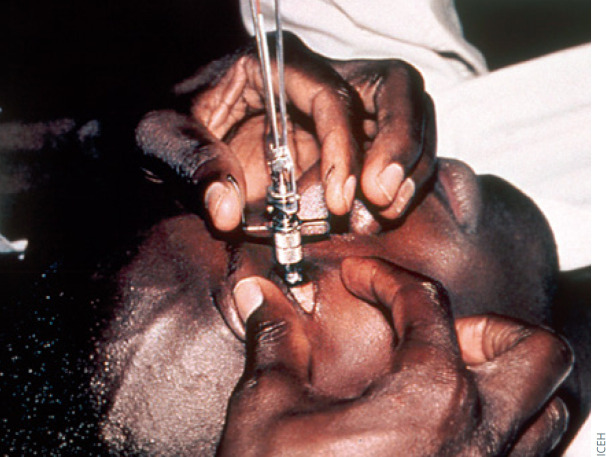
Gently place the plunger of the Schiötz tonometer on the central cornea

### You will need

- Schiötz tonometer, weights, and scale card
- Local anaesthetic drops
- Clean cotton wool or gauze swabs
- Isopropyl alcohol (70%), methylated spirit or ready-to-use alcohol wipes

### Preparation

- Test the tonometer using the spherical mould in the box and the 5.5 g weight. The pointer should swing to ‘0’ immediately (Figure 2).
- Clean the plunger and disc of the tonometer with a gauze or cotton wool swab and the isopropyl alcohol, methylated spirit or alcohol wipes. Wipe dry with a clean dry gauze or cotton wool swab.
- Lie the patient flat with her or his head supported on a pillow.

### Method

- Wash and dry your hands.
- Position yourself correctly: stand upright, behind the head of the patient, with your hands level with the patient's head. Note the health worker's good posture in Figure 3. Bad posture can affect the reading.
- Instil local anaesthetic eye drops and wait about 30 seconds.
- Ask the patient to look at a fixed object directly above the eyes. The patient's own thumb or finger held directly in front of his or her eyes) and to keep absolutely still.
- With the thumb and index finger of one hand, gently hold open the patient's eyelids, taking care not to put any pressure on the eye (see Figure 4).
- With the other hand, hold the tonometer (with the 5.5 g weight) between the thumb and index finger and place the plunger on the central cornea (see Figure 4).
- Allow the disc to lower gently onto the corneal surface.
- Note the scale reading.
- If the scale reading is ‘2’ or less, remove the tonometer, replace the 5 g weight with the 7.5 g weight and repeat the procedure.
- Note the scale reading again and remove the tonometer.
- Tell the patient not to rub the eye – the anaesthetic will last for about five minutes.
- Clean and dry the tonometer head.
- Repeat the whole procedure for the other eye.
- Clean and dry the tonometer again and store it safely in the box.
- Using the scale card, convert the noted scale readings and record the pressure in the patient's records.

**Table 1:** Scale card for ocular pressure

Scale reading	Ocular pressure, mm HG		
	5.5 g weight	7.5 g weight	10.0 g weight
**3.0**	24.4	35.8	50.6
**4.0**	20.6	30.4	43.4
**5.0**	17.3	25.8	37.2
**6.0**	14.6	21.9	31.8
**7.0**	12.2	18.5	27.2
**8.0**	10.2	15.6	23.1
**9.0**	8.5	13.1	19.6
**10.0**	7.1	10.9	16.5

## References

[1] IABP Essential List: Glaucoma. **https://iapb.standardlist.org/essential-lists/essential-list-glaucoma/**

[2] Philippin H Shah P Burton M. Detecting possible glaucoma with only limited equipment: a crucial first step. Comm Eye Health Vol. 25 No. 79 & 80 2012 pp 48 – 49. **https://cehjournal.org/article/detecting-possible-glaucoma-with-only-limited-equipment-a-crucial-first-step/**PMC3588123 23520415

[3] Baum J Chaturvedi N Netland PA Dreyer EB. Assessment of intraocular pressure by palpation. Am J Ophthalmol 1995;119(5):650–1. 7733191 10.1016/s0002-9394(14)70227-2

[4] Stevens S. How to measure intraocular pressure: Schiötz tonometry. Comm Eye Health J 2008;21(66):44. **https://cehjournal.org/article/how-to-measure-intraocular-pressure-schiotz-tonometry/**PMC2467471 18670603

